# Enhancing Stereocomplexation Ability of Polylactide by Coalescing from Its Inclusion Complex with Urea

**DOI:** 10.3390/polym9110592

**Published:** 2017-11-09

**Authors:** Ping Liu, Xiao-Tong Chen, Hai-Mu Ye

**Affiliations:** 1 Department of Materials Science and Engineering, China University of Petroleum, Beijing 102249, China; 18811414503@163.com (P.L.); chenxt_mkrc@163.com (X.-T.C.); 2 Beijing Key Laboratory of Failure, Corrosion and Protection of Oil/Gas Facilities, China University of Petroleum, Beijing 102249, China

**Keywords:** polylactide, urea, inclusion complex, crystallization, stereocomplex

## Abstract

In this study, polylactide/urea complexes were successfully prepared by the electrospinning method, then the host urea component was removed to obtain a coalesced poly(l-lactide) (PLLA)/poly(d-lactide) (PDLA) blend. The crystallization behavior of the coalesced PLLA/PDLA blend (*c*-PLLA/PDLA) was studied by a differential scanning calorimeter (DSC) and Fourier transform infrared (FTIR) spectroscopy. The *c*-PLLA/PDLA was found to show better crystallization ability than normal PLLA/PDLA blend (*r*-PLLA/PDLA). More interestingly, the *c*-PLLA/PDLA effectively and solely crystallized into stereocomplex crystals during the non-isothermal melt-crystallization process, and the reason was attributed to the equally-distributing state of PLLA and PDLA chains in the PLLA/PDLA/urea complex, which led to good interconnection between PLLA and PDLA chains when the urea frameworks were instantly removed.

## 1. Introduction

Polylactide (PLA) has become one of the most important and commercial biodegradable polymers because it possesses comparable mechanical and thermal properties with polyolefin materials [1]. However, due to the rigid chain structure and relative high glass transition temperature, it shows many drawbacks, such as a low crystallization rate and poor heat resistance; subsequently, the practical applications of PLA are remarkably restricted [2,3,4]. Thus, various methods have been used to improve the performance of PLA, including chain modification [5,6], blends with other polymers [7,8,9], and the introduction of efficient nucleating agents [10,11,12,13]. In addition to these, stereocomplex crystals formed between two PLA enantiomers, and poly(l-lactide) (PLLA) and poly(d-lactide) (PDLA) have become the most attractive selections [14,15]. The stereocomplex crystals show stronger inter-chain interaction, have a much higher melting point (~225 °C), and display a faster crystallization rate than homocrystallite of either PLLA or PDLA [16,17,18], resulting in higher mechanical strength and modulus, better heat resistance, etc. [19,20,21]. Therefore, plenty of research has been carried out. However, the formations of the stereocomplex and homocrystallite are competing during the crystallization process in the PLLA/PDLA blend [22,23]. Especially, the formation of the stereocomplex can be sharply inhibited as the PLA molecular weight increases [24,25,26]. The intrinsic occupied space leads to the steric repulsion effect among different chains, which goes against the contact between PLLA and PDLA segments, and benefits the formation of homocrystallite. In fact, only high molecular weight PLA can possess useful mechanical properties for thermoplastic applications, so several methods are employed to enhance the formation of the stereocomplex through promoting the mixed degree between PLLA and PDLA segments, such as chain topology regulation [27,28,29], addition of a compatibility agent [30,31], and the specific design of the process [32].

An inclusion complex formed between guest polymer chains and host small molecular frameworks provides a novel method to isolate polymer chains from each other in small channels [33], which offer us an effective means to obtain polymer blends with better miscibility or polymer materials with less entanglement through removing the host molecules. Until now, Tonelli’s group has carried out milestone work in this field [34,35,36,37,38,39,40]. For example, they successfully achieved intimately compatible polymer blends from normally immiscible polymers, including a PLLA/poly(ε-caprolactone) (PCL) blend and a nylon 6/nylon 66 blend [35,40]. Additionally, they obtained coalesced PCL by washing a PCL/urea inclusion complex and found that the crystallization ability and mechanical properties of PCL are significantly improved [36,39]. Recently, based on the similar method, we have successfully prepared the extended-chain crystals of poly(butylene succinate) (PBS) under atmosphere [41]. As to PLA, Howe et al. had proven that PLLA and urea could form an inclusion complex [42,43]. This, it is wondered whether PDLA chains can be adopted equally as PLLA chains in urea frameworks? Furthermore, can the complex-coalescence method be used to promote the compatibility between PLLA and PDLA and the formation of PLA stereocomplex? In this study the PLLA/urea, PDLA/urea, and PLLA/PDLA/urea complexes were prepared by electrospinning and the crystallization behaviors of the coalesced PLLA/PDLA blend were studied in detail.

## 2. Materials and Methods

### 2.1. Materials

PLLA with *M*_w_ of 2.20 × 10^5^ and 9.15 × 10^5^ g/mol and PDLA with *M*_w_ of 2.20 × 10^5^ and 9.23 × 10^5^ g/mol were purchased from Ji’nan Daigang Biological Engineering Company (Ji’nan, China). Urea (AR grade) was obtained from Shanghai Aladdin Industrial Inc. (Shanghai, China). All reagents were used without further purification.

### 2.2. Preparation of the PLA/Urea Complex and Coalesced PLA

The solution for electrospinning was prepared by dissolving PLA and urea in hexafluoroisopropanol (HFIP) with a PLA concentration fixed at 2.5 wt %. The electrospinning process was optimized as following condition: a DC voltage of 30 kV, a collector-to-needle tip distance of 18 cm, and an inner needle diameter of 0.6 mm. The as-electrospun species was dried in a vacuum oven at room temperature for two days before sealing. The weight ratio of PLA/urea was optimally selected as 1:7 based on the maximization of the experimental melting enthalpy of the complex (as seen in Figure S1).

The coalesced PLA was obtained by washing the PLA/urea complex with sufficient deionized water three times, and methanol once, to completely remove the urea component, and then drying in vacuum at 45 °C for three days.

### 2.3. Characterizations

Non-isothermal crystallization and melting behaviors of the samples were performed on a differential scanning calorimeter (DSC, 204 F1, NETZSCH, Berlin, Germany) equipped with an intercooler as cooling system under an argon atmosphere; the heating and cooling rates were set as 10 °C/min. Fourier transformation infrared (FTIR) spectra were recorded on a Hyperion spectrometer (Bruker, Karlsruhe, Germany) by signal averaging over 32 scans in the wavenumber range of 4000~400 cm^−1^; the spectrometer was equipped with a hot stage (THMS-600, Linkam, Surrey, UK) for temperature-resolution FTIR measurement.

## 3. Results

### 3.1. Characterization of the PLA/Urea Complex

**Figure 1:**
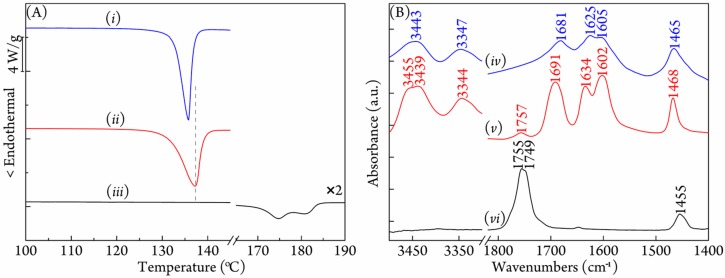
(**A**) DSC curves of (*i*) urea, (*ii*) as-prepared PLLA/urea complex and (*iii*) PLLA at a heating rate of 10 °C/min; and (**B**) the FTIR spectra of (*iv*) urea, (*v*) as-prepared PLLA/urea complex, and (*vi*) PLLA.

DSC measurement was performed to determine the formation of inclusion complex between PLLA (*M*_w_ = 2.20 × 10^5^ g/mol) and urea. As shown in Figure 1A, the as-spun product displayed a single melting point at 137.3 °C, which was different from those of urea and PLLA at 134.0 and 174.8 °C (the first melting peak), respectively. The new melting point, which was consistent with previous reports on other polymer/urea complexes, indicated the successful preparation of the PLLA/urea inclusion complex [42]. More interestingly, inclusion complexes between poly(r-3-hydroxybutyrate) (PHB) and urea, and between polypropylene and urea, showed almost the same melting point (136.8 and 138.0 °C) as PLLA/urea complex [44,45], so the melting point at around 137 °C might be a common phenomenon for the polymer/urea inclusion complexes when polymer chains contain pendant methyl groups. Figure 1B shows the FTIR spectra of in the regions from 3500 to 3300 cm^−1^ and 1820 to 1400 cm^−1^ obtained for urea, the PLLA/urea complex, and PLLA. Obviously, PLLA/urea showed quite a different FTIR spectrum from either neat urea or neat PLLA, further confirming the formation of the PLLA/urea complex. The strong N–H stretching vibration bands split and shifted from 3443 and 3347 to 3455, 3439, and 3344 cm^−1^, respectively; the C=O stretching vibration band red-shifted from 1681 to 1691 cm^−1^; the N–H bending vibration bands shifted from 1625 and 1605 to 1634 and 1602 cm^−1^; and the N–C–N antisymmetric stretching band shifted from 1465 to 1468 cm^−1^ after the urea molecules had been complexed with PLLA from its traditional tetragonal modification. As for the PLLA component in the complex, it exhibited a single C=O stretching vibration band at 1757 cm^−1^, which was quite different from the neat crystalline PLLA that had two C=O stretching vibration bands at 1755 and 1749 cm^−1^. The absence of 1749 cm^−1^ in the PLLA/urea complex revealed that PLLA chains in the complex were in an amorphous state, and the blue-shift of 2 cm^−1^, from 1755 to 1757 cm^−1^, might be due to the isolated and confined effect of the urea frameworks. The blue-shift of the C=O stretching vibration of PLLA induced by confinement had also been observed in other systems [10]. 

**Figure 2:**
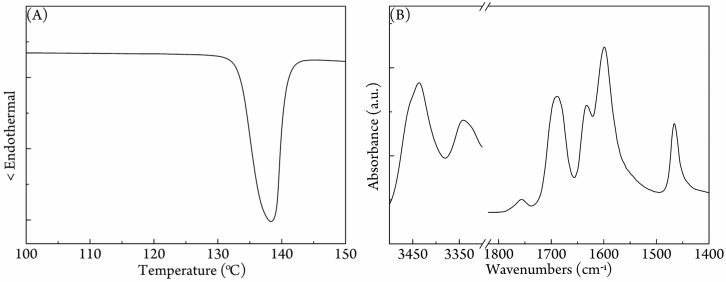
(**A**) DSC heating curve and (**B**) FTIR spectrum of the PLLA/PDLA/urea complex.

PDLA (2.2 × 10^5^ g/mol) had been also used to produce a PDLA/urea complex, and the DSC curve in Figure S2 confirms the successful preparation of PDLA/urea complex. Thus, it was expected that the guest PLLA and PDLA could be equally treated by the host urea frameworks, and equal amounts of high molecular weight PLLA (9.15 × 10^5^ g/mol) and PDLA (9.23 × 10^5^ g/mol) were employed to prepare a ternary complex with urea. Figure 2A shows that the ternary complex had a melting point of 138.3 °C; Figure 2B affirms that the ternary complex exhibited the similar FTIR spectrum as the PLLA/urea complex. Thus, the PLLA/PDLA/urea complex adopted the same structure as the PLLA/urea complex.

### 3.2. Crystallization Behavior of Coalesced PLLA/PDLA Blend

**Figure 3:**
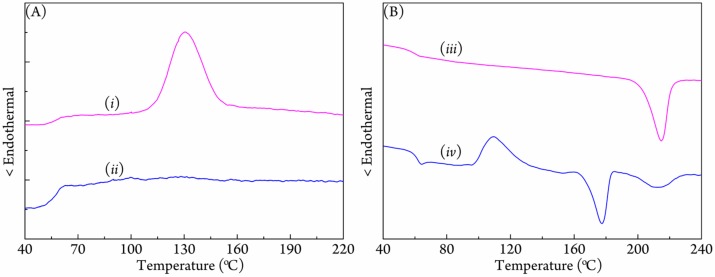
(**A**) Non-isothermal crystallization DSC curves of (*i*) coalesced PLLA/PDLA blend (*c*-PLLA/PDLA) and (*ii*) the referential PLLA/PDLA blend (*r*-PLLA/PDLA) from 240 °C; and (**B**) the subsequent DSC heating curves of (*iii*) *c*-PLLA/PDLA and (*iv*) *r*-PLLA/PDLA. Both the cooling and heating rates are 10 °C/min.

Compared with the referential PLLA/PDLA (*r*-PLLA/PDLA, prepared by the same electrospinning process without urea), the coalesced PLLA/PDLA (*c*-PLLA/PDLA) from the PLLA/PDLA/urea complex showed significant enhancement of melt-crystallization during the cooling process in Figure 3A, displaying a crystallization peak at 130.6 °C. The *r*-PLLA/PDLA did not show crystallization under the same cooling setting. During the subsequent heating (seen in Figure 3B), the *c*-PLLA/PDLA presented a single melting point at 214.9 °C, besides the normal glass transition, which demonstrated that only the stereocomplex formed between PLLA and PDLA forms during the previous cooling process. Nevertheless, a rather complicated DSC curve, which contained one exothermal peak at 109.2 °C and two endothermal peaks at 177.5 and 212.2 °C after the glass transition appeared for *r*-PLLA/PDLA; the three peaks corresponded to the cold crystallization, melt of homocrystallites of PLA, and melt of the stereocomplex of PLA, respectively. On the basis of the enthalpies of peaks at 177.5 and 212.2 °C, it is clear that PLA chains predominately cold-crystallized to homocrystals even during the heating process, which had been observed in other high molecular weight PLLA/PDLA blends [24,25,26,27,28,29,30,31,32]. Therefore, the combining processes of the PLA/urea complexation and coalescent greatly promoted the stereocomplexation ability between PLLA and PDLA. Due to the equal accommodation of PLA chains in urea frameworks, PLLA and PDLA chains would pack densely and realize an even distribution in statistics when the urea molecules were instantaneously removed by washing, which helped promote the inter-contact between PLLA and PDLA and then accelerated the stereocomplexation efficiency.

### 3.3. FTIR Study on Stereocomplexation Ability

**Figure 4:**
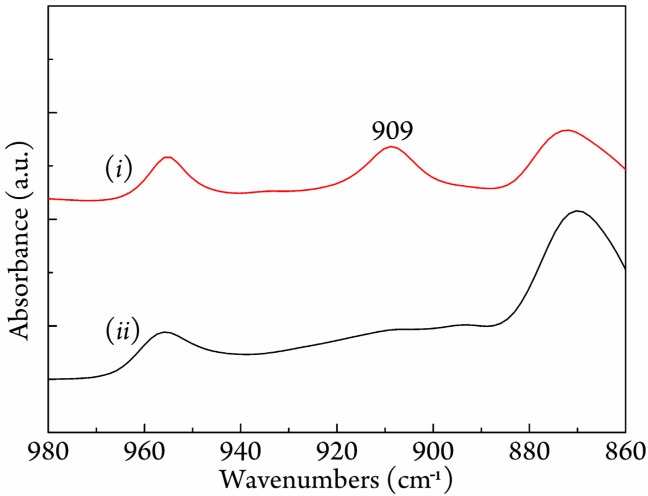
FTIR spectra of melt-quenched (*i*) *c*-PLLA/PDLA and (*ii*) *r*-PLLA/PDLA in the range from 980 to 860 cm^−1^.

To understand more about the enhancing crystallization ability for *c*-PLLA/PDLA, FTIR spectra were employed to characterize the melt-quenched sample and melting process. Figure 4 shows the FTIR spectra of *c*-PLLA/PDLA and *r*-PLLA/PDLA in a wavenumber range from 980 to 860 cm^−1^, in which a characteristic band around 909 cm^−1^ responds to the vibration mode of the *3*_1_ helical conformation in the stereocomplex [46]. The *r*-PLLA/PDLA displayed a very weak absorption band at 909 cm^−1^ when melt-quenched from 240 °C and no absorption band at 920 cm^−1^, which is the characteristic band of homocrystallite [47], indicating that the PLA chains were almost amorphous and only a small amount transformed to stereocomplex crystals. However, a rather obvious absorption band at 909 cm^−1^ appeared in the melt-quenched *c*-PLLA/PDLA specimen, meaning that the stereo-crystallization occurred remarkably.

**Figure 5:**
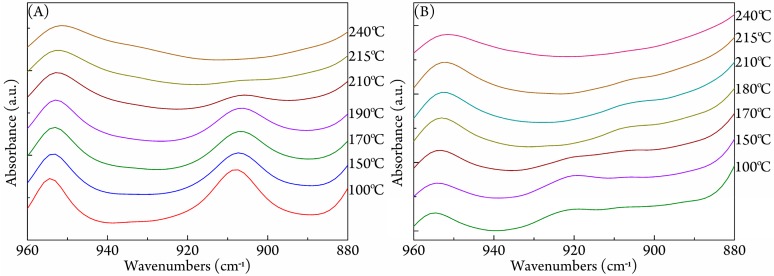
The temperature-dependent FTIR spectra of (**A**) *c*-PLLA/PDLA and (**B**) *r*-PLLA/PDLA during the heating process.

The subsequent temperature-dependent FTIR spectra of *c*-PLLA/PDLA are presented in Figure 5A. The characteristic absorption band of the *3*_1_ helical conformation in the stereocomplex at 909 cm^−1^ remained almost constant until 190 °C, then weakened at higher temperatures (i.e., 210 and 215 °C), and finally disappeared at 240 °C. For comparison, the temperature-dependent FTIR spectra of *r*-PLLA/PDLA were collected and are lined in Figure 5B. When heated to 100 °C, a characteristic band of homocrystallites rose at 920 cm^−1^, while the intensity of the 909 cm^−1^ band was still rather weak. The intensity of 920 cm^−1^ band further increased until 150 °C, then decreased, and finally disappeared at 180 °C, confirming the endothermal peak in Figure 4B originated from the cold-crystallization of homocrystallites. The further heating process after melting of homocrystals led to the formation of some sterocomplex (i.e., 180 and 210 °C), but the amount was small; and all crystallites melted at 240 °C. Both the *c*-PLLA/PDLA and the *r*-PLLA/PDLA crystallites melted and the *3*_1_ helical conformations disappeared completely at 240 °C, so the remarkable promotion of stereocomplexation in *c*-PLLA/PDLA should be due to the better inter-contacting state between PLLA and PDLA chains, which was usually rather poor in high molecular weight PLLA/PDLA blends [24,25,26]. The isolated and extended state of PLA chains in the complex would facilitate the interconnection between PLLA and PDLA during coalescing process, leading to enhancing the formation of the stereocomplex.

## 4. Conclusions

In this research, a complexation-coalescent method was employed to enhance the stereocomplexation ability of PLA. The crystallization ability of high molecular weight *c*-PLLA/PDLA was found to be stronger than *r*-PLLA/PDLA. Furthermore, the *c*-PLLA/PDLA solely formed stereocomplex crystals during the non-isothermal cooling process, and the reason was ascribed to the equal distribution of PLLA and PDLA in the PLLA/PDLA/urea complex, which led to good interconnection between PLLA and PDLA chains when the urea frameworks were instantly removed.

## Supplementary Materials

The following are available online at http://www.mdpi.com/2073-4360/9/11/592/s1; Figure S1: Dependencies of melting points and enthalpy of the complex on the PLA mass fraction; Figure S2: DSC heating curve of the PDLA/urea complex.

## References

[1] Chen G.Q., Patel M.K. (2012). Plastics Derived from Biological Sources: Present and Future: A Technical and Environmental Review. Chem. Rev..

[2] Cicero J.A., Dorgan J.R., Garrett J., Runt J., Lin J.S. (2002). Effects of molecular architecture on two-step, melt-spun poly(lactic acid) fibers. J. Appl. Polym. Sci..

[3] Drieskens M., Peeters R., Mullens J., Lemstra P.J., Hristova-Bogaerds D.G. (2009). Structure versus properties relationship of poly(lactic acid). I. Effect of crystallinity on barrier properties. J. Polym. Sci. Part B Polym. Phys..

[4] Wang L., Wang Y.N., Huang Z.G., Weng Y.X. (2015). Heat resistance, crystallization behavior, and mechanical properties of polylactide/nucleating agent composites. Mater. Des..

[5] Courgneau C., Domenek S., Lebossé R., Guinault A., Avérous L., Ducruet V. (2012). Effect of crystallization on barrier properties of formulated polylactide. Polym. Int..

[6] Loiola L.M.D., Más B.A., Duek E.A.R., Felisberti M.I. (2015). Amphiphilic multiblock copolymers of PLLA, PEO and PPO blocks: Synthesis, properties and cell affinity. Eur. Polym. J..

[7] Lu J., Qiu Z., Yang W. (2007). Fully biodegradable blends of poly(l-lactide) and poly(ethylene succinate): Miscibility, crystallization, and mechanical properties. Polymer.

[8] Pan P., Shan G., Bao Y. (2014). Enhanced nucleation and crystallization of poly(l-lactic acid) by immiscible blending with poly(vinylidene fluoride). Ind. Eng. Chem. Res..

[9] Zhao Y., Qiu Z. (2015). Effect of poly(vinyl alcohol) as an efficient crystallization-assisting agent on the enhanced crystallization rate of biodegradable poly(l-lactide). RSC Adv..

[10] Ye H.M., Hou K., Zhou Q. (2016). Improve the thermal and mechanical properties of poly(l-lactide) by forming nanocomposites with pristine vermiculite. Chin. J. Polym. Sci..

[11] Bai H.W., Huang C.M., Xiu H., Zhang Q., Fu Q. (2014). Enhancing mechanical performance of polylactide by tailoring crystal morphology and lamellae orientation with the aid of nucleating agent. Polymer.

[12] Barrau S., Vanmansart C., Moreau M., Addad A., Stoclet G., Lefebvre J.M., Seguela R. (2011). Crystallization behavior of carbon nanotube-polylactide nanocomposites. Macromolecules.

[13] Zhao L., Liu X., Zhang R., He H.F., Jin T., Zhang J. (2015). Unique morphology in polylactide/graphene oxide nanocomposites. J. Macromol. Sci. Phys..

[14] Ikada Y., Jamshidi K., Tsuji H., Hyon S.H. (1987). Stereocomplex formation between enantiomeric poly(lactides). Macromolecules.

[15] Tsuji H. (2005). Poly(lactide) stereocomplexes: Formation, structure, properties, degradation, and applications. Macromol. Biosci..

[16] Schmidt S.C., Hillmyer M.A. (2001). Polylactide stereocomplex crystallites as nucleating agents for isotactic polylactide. J. Polym. Sci. Part B Polym. Phys..

[17] Tsuji H., Tezuka Y. (2004). Stereocomplex formation between enantiomeric poly(lactic acid)s. 12. Spherulite growth of low-molecular-weight poly(lactic acid)s from the melt. Biomacromolecules.

[18] Tsuji H., Takai H., Saha S.K. (2006). Isothermal and non-isothermal crystallization behavior of poly(l-lactic acid): Effects of stereocomplex as nucleating agent. Polymer.

[19] Tsuji H., Ikada Y. (1999). Stereocomplex formation between enantiomeric poly(lactic acid)s. XI. Mechanical properties and morphology of solution-cast films. Polymer.

[20] Ma P.M., Shen T.F., Xu P.W., Dong W.F., Lemstra P.J., Chen M.Q. (2015). Superior performance of fully biobased poly(lactide) via stereocomplexation-induced phase separation: Structure versus property. ACS Sustain. Chem. Eng..

[21] Tsuji H., Fukui I. (2003). Enhanced thermal stability of poly(lactide)s in the melt by enantiomeric polymer blending. Polymer.

[22] Tsuji H., Hyon S.H., Ikada Y. (1991). Stereocomplex formation between enantiomeric poly(lactic acid)s. 3. Calorimetric studies on blend films cast from dilute solution. Macromolecules.

[23] Tsuji H., Ikada Y. (1993). Stereocomplex formation between enantiomeric poly(lactic acids). 9. Stereocomplexation from the melt. Macromolecules.

[24] Pan P.J., Han L.L., Bao J.N., Xie Q., Shan G.R., Bao Y.Z. (2015). Competitive stereocomplexation, homocrystallization, and polymorphic crystalline transition in poly(l-lactic acid)/poly(d-lactic acid) racemic blends: Molecular weight effects. J. Phys. Chem. B.

[25] Han L.L., Pan P.J., Shan G.R., Bao Y.Z. (2015). Stereocomplex crystallization of high-molecular-weight poly(l-lactic acid)/poly(d-lactic acid) racemic blends promoted by a selective nucleator. Polymer.

[26] Tsuji H., Tashiro K., Bouapao L., Hanesaka M. (2012). Synchronous and separate homo-crystallization of enantiomeric poly(l-lactic acid)/poly(d-lactic acid) blends. Polymer.

[27] Han L.L., Shan G.R., Bao Y.Z., Pan P.J. (2015). Exclusive stereocomplex crystallization of linear and multiarm star-shaped high-molecular-weight stereo diblock poly(lactic acid)s. J. Phys. Chem. B.

[28] Han L.L., Yu C.T., Zhou J., Shan G.R., Bao Y.Z., Yun X.Y., Dong T., Pan P.J. (2016). Enantiomeric blends of high-molecular-weight poly(lactic acid)/poly(ethylene glycol) triblock copolymers: Enhanced stereocomplexation and thermomechanical properties. Polymer.

[29] Fukushima K., Kimura Y. (2008). An efficient solid-state polycondensation method for synthesizing stereocomplexed poly(lactic acid)s with high molecular weight. J. Polym. Sci. Part A Polym. Chem..

[30] Li S.H., Woo E.M. (2009). Kinetic Analysis on Effect of Poly(4-vinyl phenol) on Complex-Forming Blends of Poly(l-lactide) and Poly (d-lactide). Polym. J. Tokyo Jpn..

[31] Pan P.J., Bao J.N., Han L.L., Xie Q., Shan G.R., Bao Y.Z. (2016). Stereocomplexation of high-molecular-weight enantiomeric poly(lactic acid)s enhanced by miscible polymer blending with hydrogen bond interactions. Polymer.

[32] Bao R.Y., Yang W., Jiang W.R., Liu Z.Y., Xie B.H., Yang M.B., Fu Q. (2012). Stereocomplex formation of high-molecular-weight polylactide: A low temperature approach. Polymer.

[33] Lu J., Mirau P.A., Tonelli A.E. (2002). Chain conformations and dynamics of crystalline polymers as observed in their inclusion compounds by solid-state NMR. Prog. Polym. Sci..

[34] Gurarslan A., Joijode A.S., Tonelli A.E. (2012). Polymers Coalesced from Their Cyclodextrin Inclusion Complexes: What Can They Tell Us about the Morphology of Melt-Crystallized Polymers?. J. Polym. Sci. Part B Polym. Phys..

[35] Wei M., Shuai X., Tonelli A.E. (2003). Melting and crystallization behaviors of biodegradable polymers enzymatically coalesced from their cyclodextrin inclusion complexes. Biomacromolecules.

[36] Williamson B.R., Krishnaswamy R., Tonelli A.E. (2011). Physical properties of poly(ε-caprolactone) coalesced from its α-cyclodextrin inclusion compound. Polymer.

[37] Gurarslan A., Shen J., Tonelli A.E. (2012). Behavior of poly(ε-caprolactone)s (PCLs) coalesced from their stoichiometric urea inclusion compounds and their use as nucleants for crystallizing PCL melts: Dependence on PCL molecular weights. Macromolecules.

[38] Wei M., Davis W., Urban B., Song Y., Porbeni F.E., Wang X., White J.L., Balik C.M., Rusa C.C., Fox J., Tonelli A.E. (2002). Manipulation of Nylon-6 Crystal Structures with Its α-Cyclodextrin Inclusion Complex. Macromolecules.

[39] Gurarslan A., Caydamli Y., Shen J.L., Tse S., Yetukuri M., Tonelli A.E. (2015). Coalesced poly(ε-caprolactone) fibers are stronger. Biomacromolecules.

[40] Wei M., Shin I.D., Urban B., Tonelli A.E. (2004). Partial miscibility in a nylon-6/nylon-66 blend coalesced from their common α-cyclodextrin inclusion complex. J. Polym. Sci. Part B Polym. Phys..

[41] Ye H.M., Chen X.T., Liu P., Wu S.Y., Jiang Z.Y., Xiong B.J., Xu J. (2017). Preparation of Poly(butylene succinate) Crystals with Exceptionally High Melting Point and Crystallinity from Its Inclusion Complex. Macromolecules.

[42] Howe C., Vasanthan N., MacClamrock C., Sanker S., Skin I.D., Simonsen I.K., Tonelli A.E. (1994). Inclusion compound formed between poly(l-lactic acid) and urea. Macromolecules.

[43] Howe C., Sankar S., Tonelli A.E. (1993). ^13^C n.m.r observation of poly (l-lactide) in the narrow channels of its inclusion compound with urea. Polymer.

[44] Eaton P., Vasanthan N., Shin I.D., Tonelli A.E. (1996). Formation and Characterization of Polypropylene-Urea Inclusion Compounds. Macromolecules.

[45] Ravindran P., Vasanthan N. (2015). Formation of Poly(3-hydroxybutyrate) (PHB) Inclusion Compound with Urea and Unusual Crystallization Behavior of Coalesced PHB. Macromolecules.

[46] Okihara T., Tsuji M., Kawaguchi A., Katayama K.I., Tsuji H., Hyon S.H., Ikada Y. (1991). Crystal structure of stereocomplex of poly(l-lactide) and poly(d-lactide). J. Macromol. Sci. Part B Phys..

[47] Kister G., Cassanas G., Vert M., Pauvert B., Térol A. (1995). Vibrational analysis of poly(l-lactic acid). J. Raman Spectrosc..

