# Supplementary material for: Enhancing Stereocomplexation Ability of Polylactide by Coalescing from Its Inclusion Complex with Urea
Source: Polymers (Basel). 2017 Nov 9;9(11):592. doi: 10.3390/polym9110592 (PMC6418699; doi:10.3390/polym9110592)
Supplement: Supplementary file 1 [file polymers-09-00592-s001.pdf]

Supplementary Materials: Enhancing Stereocomplexation Ability of Polylactide by Coalescing from Its Inclusion Complex with Urea

Ping Liu, Xiao-Tong Chen and Hai-Mu Ye

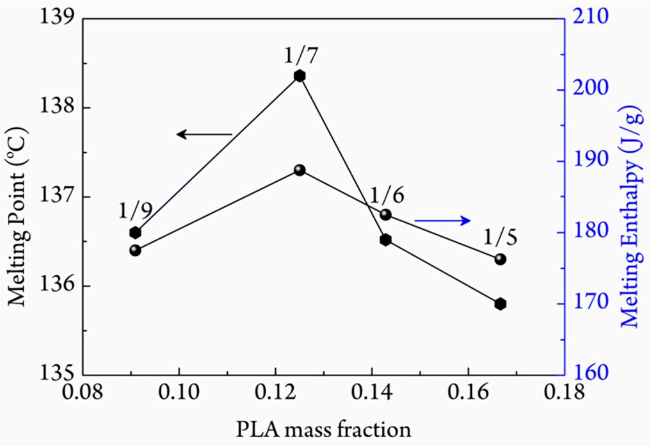

Figure S1. The isothermal crystallization curves of (**A**) *c*-PLLA and (**B**) *r*-PLLA at different temperatures.

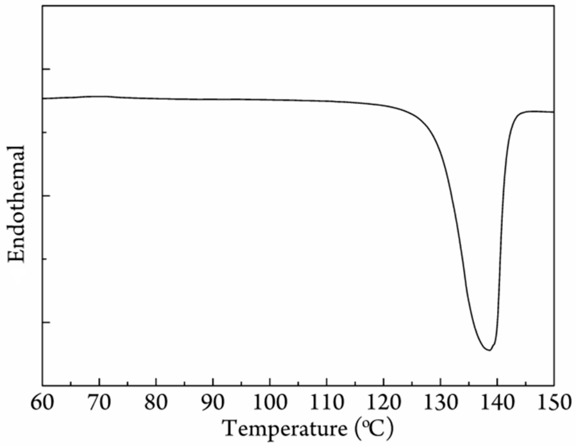

Figure S2. DSC heating curve of PDLA/urea complex.
